# Hydrodynamics of a cold circulating fluidized bed for methanol-to-olefins (MTO) process: experimental and computational investigation

**DOI:** 10.1038/s41598-023-45326-6

**Published:** 2023-10-26

**Authors:** Mojtaba Babaei, Salman Movahedirad

**Affiliations:** School of Chemical Engineering, Iran University of Science and Technology, Tehran, Iran

**Keywords:** Chemical engineering, Computational science

## Abstract

The present study aims to investigate the hydrodynamic behavior of gas–solid flow in a pseudo-two-dimensional cold circulation fluidized bed specifically designed to mimic a plane cut of an industrial methanol-to-olefins (MTO) bed. The solid velocity pattern was experimentally examined using the Particle Image Velocimetry with Digital Image Analysis (PIV-DIA) technique. The hydrodynamics of this pseudo-2D bed were studied using the twoPhaseEulerFoam solver in OpenFOAM, and the adjusted solver was validated through solid particle velocity and solid volume fraction comparisons, showing good agreement with experimental data. Additionally, the study explores different geometries, including symmetric and asymmetric baffles, to understand their impact on the mixing characteristics of the circulating fluidized bed for MTO. The findings highlight the significant role of asymmetric baffles in enhancing mixing behavior. Furthermore, the DIA technique was employed to study the effects of bubbles on gas–solid mixing, revealing that the low mixing region of the bed limited the diameter of bubbles.

## Introduction

The demand for ethane and propane as the two most common olefins has been steadily increasing, underscoring the importance of modifying related production processes. Among the widely employed methods for olefin production, catalytic conversion of methanol to olefins (MTO) stands out as a prominent approach. Methanol, an intermediate product of the petrochemical industry, can be obtained from coal or natural gas, making the MTO process a crucial link between the gas/coal industry and modern petrochemical complexes^1,2^. Extensive research has identified the circulating fluidized bed reactor as a promising option for MTO reactions^2–5^. These reactors are favored in the petroleum refinery industries due to their catalyst-regeneration capabilities and enhanced mass/heat transfer rates^6,7^. Nevertheless, a comprehensive understanding of the transport phenomena within the reactor is imperative for effective design and scale-up of circulating fluidized bed reactors^8,9^.

To gain insights into the hydrodynamics of gas–solid fluidized beds, researchers have employed both numerical and experimental methods^10–16^. Numerical simulations have predominantly adopted two approaches: Eulerian–Eulerian (Eu–Eu) and Eulerian–Lagrangian (Eu–La) methods. Eu–Eu methods treat the fluid and solid phases as interpenetrating media, solving separate Navier-Stocks equations for each phase and modeling momentum exchange through various drag force equations^17^. In contrast, Eu–La methods account for the discrete nature of solid particles by employing Newtonian motion equations^10–14,18^.

In our research, the choice of drag model was a crucial aspect of our simulation methodology. We conducted a careful evaluation of various drag models available in the literature, guided by the recent research conducted by Li et al.^19^. Their study systematically assessed the performance of different drag models in predicting gas–solid hydrodynamics in fluidized beds. Their findings revealed that, when considering the distribution of time-averaged particle axial velocity in the y-direction, most drag models yielded results consistent with experimental and Direct Numerical Simulation (DNS) data. However, it's important to note that exceptions were observed for the Wen-Yu and Tenneti-Garg-Subramaniam (TGS) models. Therefore, in the literature, various drag models such as Gidaspow or EEM have been used to simulate the circulating fluidized bed reactors^7,20,21^.

Upon comparing our simulation results with experimental data, we observed that the Gidaspow drag model provided accurate predictions. This alignment between our findings and experimental outcomes led us to select the Gidaspow drag model for our research, ensuring that our numerical simulations accurately capture the hydrodynamics of gas–solid fluidized beds.

Experimental methods for evaluating gas–solid fluidized bed behavior include particle image velocimetry (PIV), positron emission, X-ray techniques, among others^5,22–27^. Given the very short residence time of gas within the MTO reactor (approximately 2–3 s), achieving efficient contact of reactants (gas phase) with fresh catalysts becomes crucial. However, industrial circulating fluidized bed reactors often exhibit incomplete mixing, resulting in a wide residence time distribution for solid particles^2^. Thus, an in-depth investigation of the mixing behavior of fresh and coked particles is essential^28^.

While various studies have validated their numerical simulations based on product concentrations, surprisingly, there is a lack of published works that experimentally investigate circulating fluidized bed reactors for MTO and validate the numerical simulations of their hydrodynamics^12,29^. Considering that enhanced gas–solid mixing inside fluidized bed reactors can significantly influence reaction rates^12,29^, a thorough understanding of the averaged velocity trends of particles becomes a prerequisite before examining kinetic effects.

The current investigation aims to shed light on the mixing characteristics of a non-riser circulating fluidized bed reactor, providing valuable insights for the development of an industrial MTO fluidized bed reactor. To achieve this, we studied the solid flow pattern in a pseudo-2D cold circulating fluidized bed using the Particle Image Velocimetry with Digital Image Analysis (PIV-DIA) technique. Notably, an area with a high solid volume fraction and slow movement was observed, indicating incomplete solid mixing. Furthermore, we explored the hydrodynamics of the MTO process within the circulating fluidized bed using the twoPhaseEulerFoam solver in OpenFOAM. Our simulations investigated the impact of different baffle geometries on the mixing behavior of the bed, especially focusing on mitigating dead zones that could reduce bed efficiency.

In summary, this investigation addresses a critical knowledge gap by thoroughly examining the hydrodynamics of a non-riser circulating fluidized bed reactor for MTO. By combining experimental and numerical approaches, we aim to improve the understanding of gas–solid mixing and provide essential insights for the development of efficient industrial MTO fluidized bed reactors.

## Experimental

### Experimental set-up

**Figure 1:**
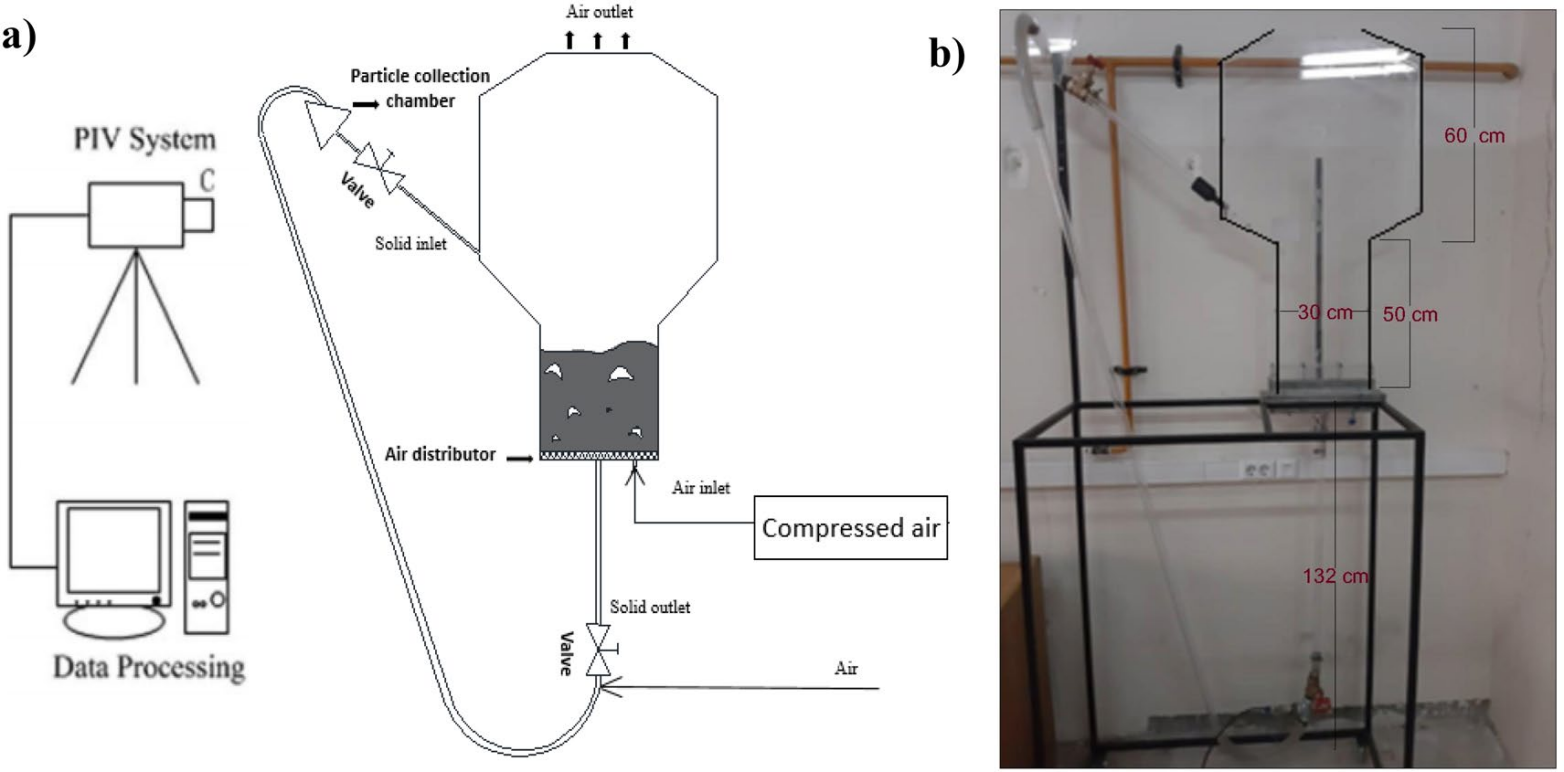
The two-dimensional circulating fluidized bed set-up. (**a**) schematic diagram (**b**) real set-up.

In the study of gas–solid flow hydrodynamics, particularly in fluidized bed reactors, the volume fraction and velocity field of solid particles are critical parameters. Additionally, knowing the hydrodynamic behavior of fluidized beds is necessary for reliable two-phase flow simulation. As the study utilized an image-based measurement technique, a two-dimensional set-up comprising of transparent Plexiglass sheets was utilized. Figure 1 depicts the pseudo-2D circulating fluidized bed set-up, which comprises of two zones with distinct cross-sectional areas. The reaction zone and sedimentation zone correspond to the lower and upper sections, respectively. The geometry of this setup are in line with those of the industrial pilots used in the MTO process^2^.

The wind box serves as the entry point for inlet air from the bottom of the bed. The air then flows through a stationary bed of porous particles, ensuring uniform distribution before entering the bed. After passing through the bed, the air exits above it, and no particles are present in the outlet air. The design incorporates a standpipe compose of three section and a particle collection chamber. As solid particles in the fluidized bed move downward, they pass through the vertical standpipe and are then directed by a secondary airflow to the particle collection chamber through the upward section of the standpipe, as depicted in Fig. 1. To regulate the solid circulation rate in the bed, two slide valves are employed on both the vertical and inclined sections of the standpipe.

### Image-based measurement methods

The set-up structure provides a good platform for image-based measurements. The location of particles and bubbles in the bed is determined by high-quality imaging. Analyzing the light intensity of gray-scale images determines the volume fraction occupied by the solid phase. Additionally, to further support the image-based measurements and validation process, Table 1 presents the key parameters used during Particle Image Velocimetry and Digital Image Analysis (PIV-DIA). The utilization of high-speed imaging with the PIV technique also facilitates the analysis of particle displacements over specific time intervals, enhancing the precision and reliability of our experimental data. Moreover, high-speed imaging provides valuable insights into the displacement of particles per unit time delay through the PIV technique.

**Table 1:** PIV-DIA parameters.

Parameter	Value
Image acquisition settings	Frame rate: 120 fps
Interrogation window size	64 × 64 pixels
Overlap	50%,

#### Estimation of the volume fraction of solid phase

To determine the concentration of solid particles in a fluidized bed, the average gray-scale value of the image was analyzed using the method proposed by Gras and Abanades^30^. For calibration, the fixed bed structure was taken as the reference mode. Using the following equation, the local volume fraction of the solid phase was calculated:

1 $${\alpha }_{s}={\left({\alpha }_{s}\right)}_{ref}\frac{ln\left(\frac{{GSV}_{1}}{GSV}\right)}{ln\left(\frac{{GSV}_{1}}{{GSV}_{2}}\right)}$$

In the aforementioned equation, the gray-scale value (GSV) of a pixel is determined through the digital image analysis technique using the ImageJ software. The indices 1 and 2 correspond to the zero and maximum volume fraction calibration reference modes, respectively.

#### Particle image velocimetry (PIV)

**Figure 2:**
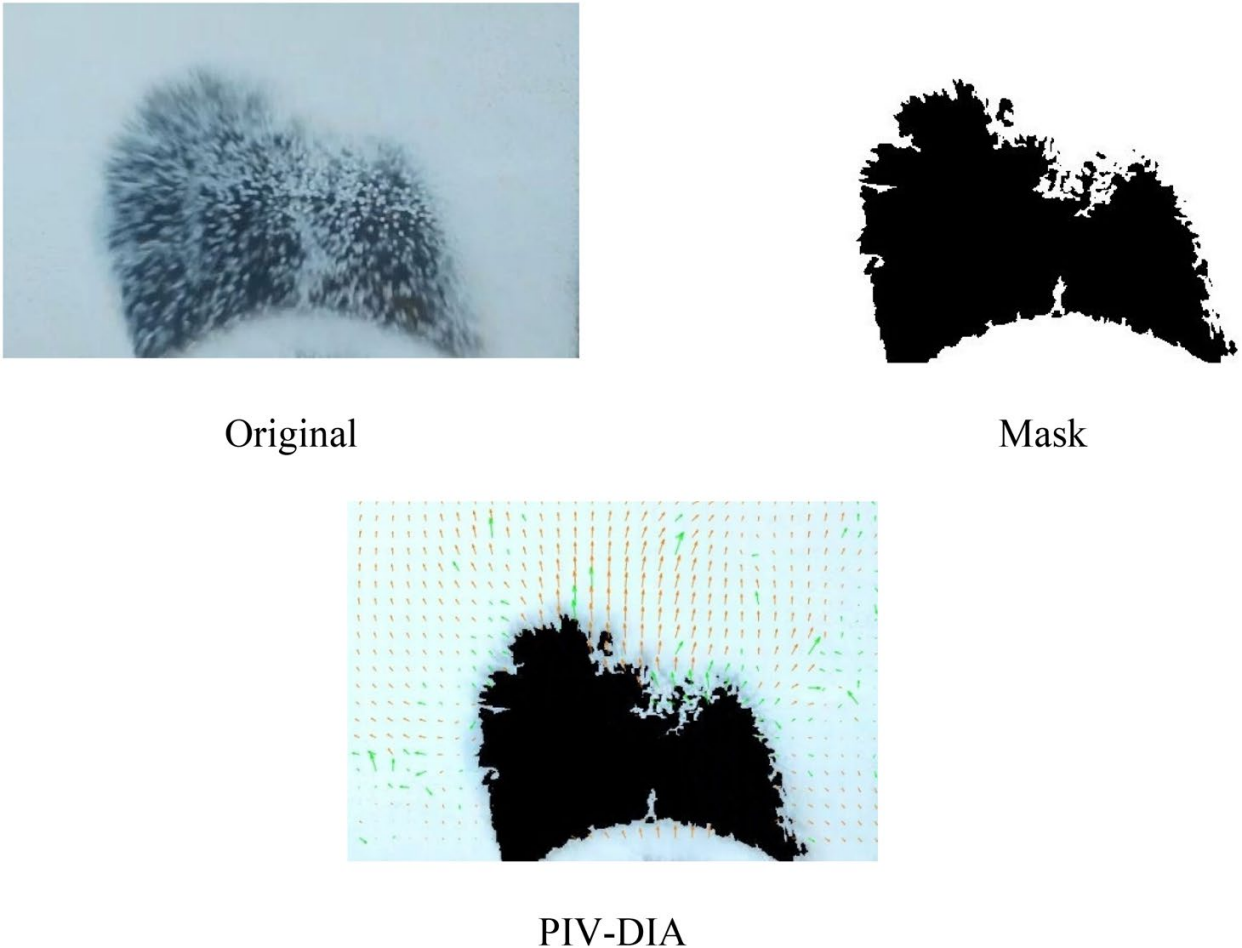
The procedure of throughput PIV-DIA images.

Particle Image Velocimetry (PIV) is a widely-used technique to visualize fluid flow fields. It works by measuring the movement of particles in a small window, and the resulting velocity field is calculated using cross-correlation between successive frames^11,13,14^. In this study, the interrogation window size was set to 64 × 32 pixels, with a 50% overlapping area to obtain a dense velocity field. However, care must be taken when using PIV to measure the flow field in fluidized beds, as fine raining particles inside the ascending bubbles can cause unrealistic and very high-speed values^8,13^, leading to significant errors in the averaged results. To address this issue, the coupled PIV-DIA method developed by previous researchers was employed, as shown in Fig. 2. In this method, the images are represented as a two-dimensional array with elements of either zero or one, where the value of zero is assigned to the bubbles. By multiplying element by element of the matrix of the original image and the matrix of the masked image, a corrected image is obtained. The time-averaged velocity profile is then produced through post-processing of the images.

#### Sampling and measurement procedure

To investigate the dominant phenomena in the sedimentation and reaction zones, high-speed photography was performed according to Fig. 1. Velocity profiles at dimension less heights of 0.1 and 0.4. To ensure accuracy and consistency with the simulation time, all image averaging was carried out throughout at least 180 s, which was found to be sufficient for averaging the velocity data.

## Numerical simulation

The gas–solid flow is commonly modeled using the Eulerian–Lagrangian or Eulerian–Eulerian approaches. The Eulerian–Lagrangian approach combines computational fluid dynamics (CFD) with the discrete element method (DEM) and provides detailed information about the motion of individual solids using Newton's second law. On the other hand, the Eulerian–Eulerian approach assumes that both the liquid and solid phases are continuous and the Eulerian–Eulerian method is frequently used to simulate dense flow in fluidized beds^20,31^, as it offers a balance between processing time and accuracy. Simulating a case with a large number of particles using Eulerian–Lagrangian solvers incurs a high computational cost, which is the most significant limiting factor. Key simulation outputs include the velocity field, volume fraction of solid particles, and granular temperature.

In the present study, an Eulerian–Eulerian approach was used by the OpenFOAM software as an open-source computational fluid dynamics tool. One of its key advantages is its flexibility, which enables users to explore all aspects of coding, including modification and development.

### Governing equations

As mentioned earlier an Eulerian–Eulerian method was employed to simulate the particle flow pattern in the MTO reactor because of its accuracy and low computational cost. In this study, a two-phase model was employed, which treats the two phases as continuous. The model accounts for the exchange of momentum and energy between the phases, but in reality, particles do not always behave as a continuous phase. To consider particle–particle interactions, the kinetic theory of granular flow (KTGF) was employed. KTGF is similar to the kinetic theory of dense gases (KTDG). In the two-fluid model, conservation equations for the gas and solid phases are used as follows:

2 $$\nabla .\left({\alpha }_{g}{\rho }_{g}{u}_{g}\right)+\frac{\partial }{\partial t}\left({\alpha }_{g}{\rho }_{g}\right)=0$$

3 $$\nabla .\left({\alpha }_{s}{\rho }_{s}{u}_{s}\right)+\frac{\partial }{\partial t}\left({\alpha }_{s}{\rho }_{s}\right)=0$$

4 $$\nabla .\left({\alpha }_{g}{\rho }_{g}{u}_{g}{u}_{g}\right)+\frac{\partial }{\partial t}\left({\alpha }_{g}{\rho }_{g}{u}_{g}\right)=-{\alpha }_{g}\nabla \mathrm{p}+\nabla .\left({\tau }_{g}{\alpha }_{g}\right){+\alpha }_{g}{\rho }_{g}g+\beta ({u}_{s}-{u}_{g})$$

5 $$\nabla .\left({\alpha }_{s}{\rho }_{s}{u}_{s}{u}_{s}\right)+\frac{\partial }{\partial t}\left({\alpha }_{s}{\rho }_{s}{u}_{s}\right)=-{\alpha }_{s}\nabla \mathrm{p}-\nabla {p}_{s}+\nabla .\left({\tau }_{s}{\alpha }_{s}\right){+\alpha }_{s}{\rho }_{s}\mathrm{g}+\beta ({u}_{g}-{u}_{s})$$

where, ${\tau }_{g}$ and ${\tau }_{s}$ represent the viscous stress of gas and solid phases, respectively:

6 $${\tau }_{g}={\mu }_{g}\left(\nabla {u}_{g}+{\nabla }^{T}{u}_{g}\right)-\frac{2}{3}{\mu }_{s}(\nabla .{u}_{g})I$$

7 $${\tau }_{s}={\mu }_{s}\left(\nabla {u}_{s}+{\nabla }^{T}{u}_{s}\right)+({\lambda }_{s}-\frac{2}{3}{\mu }_{s})(\nabla .{u}_{s})I$$

The drag coefficient was determined from the relationships developed by Gidaspow ^32^:

8 $${C}_{D}=\frac{24}{{Re}_{p}}\left(1+0.15 {{Re}_{p}}^{0.687}\right); {Re}_{p}<1000$$

9 $${C}_{D}=0.44;{ Re}_{p}\ge 1000$$

Depending on the volume fraction of the solid phase, there are two relationships for calculating $\beta$ in the literature^33^:

10 $$\beta =\frac{3}{4}\frac{{C}_{D}{\alpha }_{g}{\alpha }_{s}{\rho }_{g}\left|{u}_{g}-{u}_{s}\right|}{{d}_{p}}{{\alpha }_{g}}^{-2.65}; {\alpha }_{s}<0.2$$

11 $$\beta =150\frac{{\mu }_{s}{{\alpha }_{s}}^{2}}{{{\alpha }_{g}}^{2}{{d}_{p}}^{2}}+1.75\frac{{\rho }_{g}{\alpha }_{s}}{{\alpha }_{g}{d}_{p}}\left|{u}_{g}-{u}_{s}\right|; {\alpha }_{s}>0.2$$

The KTGF equation is formulated by considering binary collisions between idealized spherical particles and accounting for particle fluctuations in a localized region over a brief period^34^.

12 $$\frac{3}{2}\left(\frac{\partial }{\partial t}\left({\rho }_{s}{\alpha }_{s}\theta \right)+\nabla .({\rho }_{s}{\alpha }_{s}{u}_{s}\theta )\right)=\left({\tau }_{s}-{p}_{s}I\right):\nabla {u}_{s}+\nabla .\left({f}_{s}\nabla \theta \right)+{J}_{vis}+{J}_{slip}-{\gamma }_{s}$$

The granular energy equation involves several components, including the granular energy diffusion flux represented by ∇. (f_s ∇θ), and dissipation factors such as γ_s, J_vis, and J_slip. The dissipation factors correspond to inelastic collisions, fluid viscosity, and fluid turbulence, respectively.

13 $${p}_{s}={\rho }_{s}{\alpha }_{s}\theta +2{{\alpha }_{s}}^{2}{g}_{0}\theta (1+{e}_{s})$$

14 $${f}_{s}=\frac{2}{\left(1+{e}_{s}\right){g}_{0}}{\left(1+\frac{6}{5}\left(1+{e}_{s}\right){g}_{0}{\alpha }_{s}\right)}^{2}\left(\frac{75}{384}\sqrt{\pi \theta }{\rho }_{s}{d}_{p}\right)+2{{\alpha }_{s}}^{2}{\rho }_{s}{d}_{p}{g}_{0}\left(1+{e}_{s}\right)\sqrt{\frac{\theta }{\pi }}$$

15 $${J}_{vis}=3\beta \theta $$

16 $${J}_{slip}=\frac{81{\alpha }_{s}{\mu }_{g}}{{g}_{0}{{d}_{p}}^{3}{\rho }_{s}\sqrt{\pi \theta }}\left|{u}_{g}-{u}_{s}\right|\varphi $$

17 $$\varphi =\frac{{R}_{d}^{2}}{1+3.5\sqrt{{\alpha }_{s}}+5.9{\alpha }_{s}}$$

18 $$ R_{d}  = \left\{ {\begin{array}{*{20}l}    {\frac{{1 + 3\sqrt {\frac{{\alpha _{s} }}{2}}  + \frac{{135}}{{64}}\alpha _{s} {\text{ln}} \left( {\alpha _{s} } \right) + 17.4\alpha _{s} }}{{1 + 0.681\alpha _{s}  - 8.48\alpha _{s} ^{2}  + 8.16\alpha _{s} ^{3} }}\quad \alpha _{s}  < 0.4} \hfill  \\    {\frac{{10\alpha _{s} }}{{(1 - \alpha _{s} )^{3} }} + 0.7\quad\alpha _{s}  \ge 0.4} \hfill  \\   \end{array} } \right. $$

19 $${\gamma }_{s}=3(1-{{e}_{s}}^{2}){{\alpha }_{s}}^{2}{\rho }_{s}{g}_{0}\theta \left(\frac{4}{{d}_{p}}\sqrt{\frac{\theta }{\pi }}-\nabla .{u}_{s}\right)$$

20 $${g}_{0}=\frac{1}{1-{(\frac{{\alpha }_{s}}{{\alpha }_{s},max})}^\frac{1}{3}}$$

where, ${e}_{s}$ and ${g}_{0}$ are the coefficient of restitution for solids and radial distribution functions, respectively.

### twoPhaseEulerFoam solver

The twoPhaseEulerFoam solver is designed for modeling a system that comprises two compressible phases that do not undergo any chemical reactions. It was initially developed by Rusche^35^. The twoPhaseEulerFoam solves the momentum and energy equations of both phases. To model the stress tensor of the solid phase in twoPhaseEulerFoam, there are two approaches available^36^. The first approach involves an inviscid solid phase with a pressure model, while the second approach utilizes the stress tensor obtained through KTGF and frictional models.

### Geometry, boundary conditions, and simulation conditions

**Figure 3:**
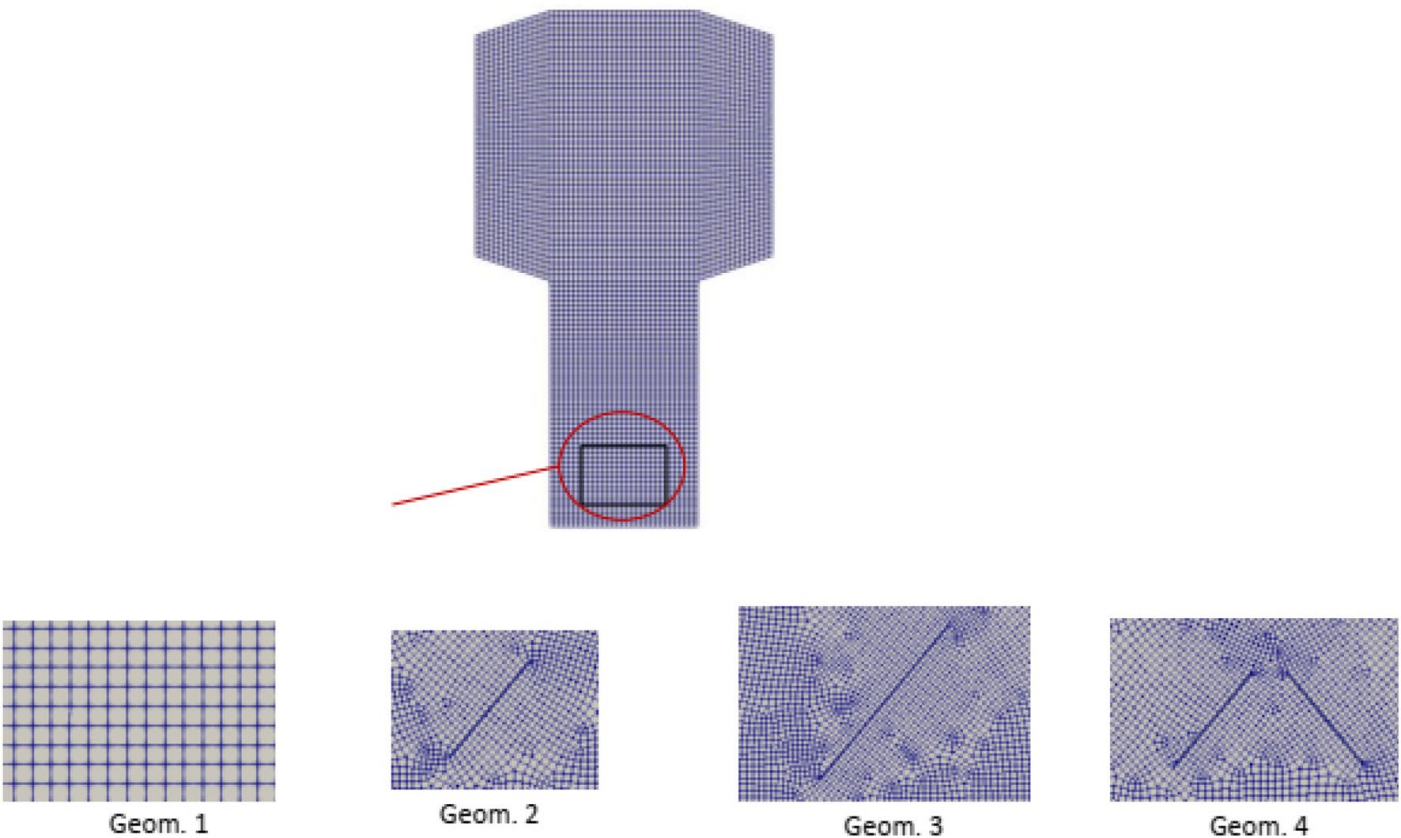
Various geometries of the system.

Four different geometries depicted in Figure 3 were discretized in two dimensions. Geom. 1 corresponds to the two-dimensional experimental setup of MTO shown in Fig. 1, but without a baffle. Geoms. 2, 3, and 4 include baffles located close to the solid flow outlet, each with a slope of approximately 50°. The length of the baffle in Geom. 3 is twice that of the baffle in Geom. 2 (i.e., 7.81 cm). Geom. 4, on the other hand, utilizes two symmetrical baffles of the same size as the baffle in Geom. 2.

Table 2 Mesh properties of the simulation provides the mesh characteristics for the different geometries. The number of grids in each case is chosen to ensure that the results are independent of the mesh size. As can be observed from Table 2, for the cases with baffles, a greater number of cells are used to capture the velocity vector field near the tilted baffles more accurately.

**Table 2:** Mesh properties of the simulation.

Case	Geom. 1	Geom. 2	Geom. 3	Geom. 4
Points	9088	55,682	43,798	39,132
Faces	17,698	110,230	86,570	77,353
Internal faces	8612	54,548	42,772	38,219
Cells	4385	27,463	21,557	19,262

**Figure 4:**
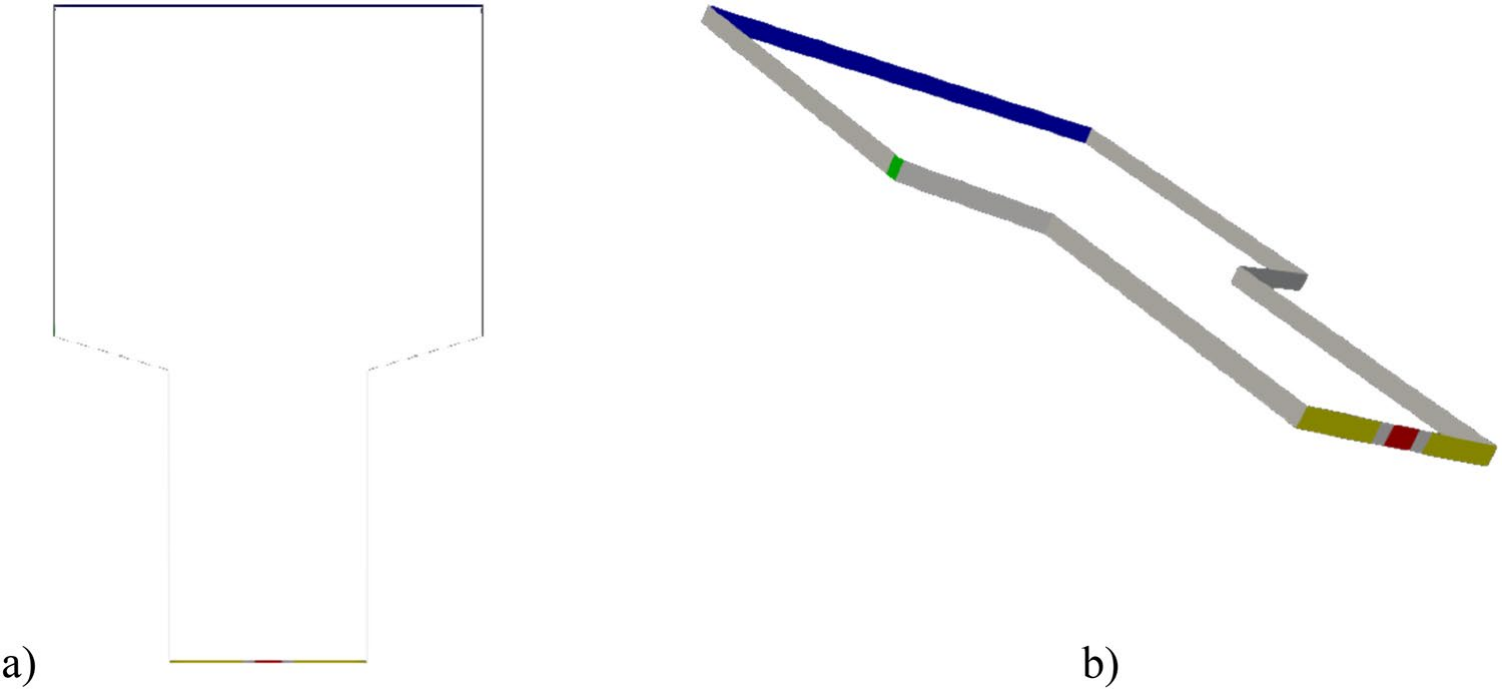
Schematic Representation of Boundary Conditions with Dual Perspectives (Viewpoints **a** and **b**)—Color-coded Legend: Gray (Walls), Blue (Gas Outlet), Yellow (Gas Inlet), Green (Solid Inlet), Red (Solid Outlet).

To provide a comprehensive representation of the boundary conditions used in our simulation employing the twoPhaseEulerFoam solver, we have included Fig. 4, a schematic diagram showcasing the specific locations of each boundary and the corresponding boundary conditions applied to them, as detailed in Table 5.

As elaborated in Tables 3, 4, and 5, we have presented extensive information on the physical properties of both the solid and gas phases, physical models, and other essential input data. To enhance the visual understanding of the boundary conditions, we have color-coded the boundaries as follows: Gray represents Walls, Blue denotes Gas outlets, Yellow indicates Gas inlets, Green designates Solid inlets, and Red represents Solid outlets.

**Table 3:** Physical properties of gas and solid phases.

Solid phase		Gas phase	
Particle mean diameter (μm)	920	Air viscosity (Pa.s)	1.84e−05
Particle density (kg/m^3^)	1095	Air density (kg/m^3^)	Ideal gas
Particle heat capacity (J/kg/K)	500,000	Air heat capacity (J/kg/K)	1007
Maximum volume fraction	0.63	Air Prandtl number	0.7

**Table 4:** Physical models for CFD-simulations.

Model type	Value
Diameter models	Constant
Drag model	Gidaspow
Turbulence models	RAS
Viscosity model	Gidaspow
Conductivity model	Gidaspow
Granular pressure model	Lun
Radial function model	SinclairJackson
Frictional stress model	JohnsonJackson

**Table 5:** The boundary condition for the CFD simulations.

	Gas velocity	Solid velocity	${\alpha }_{s}$	$\theta$
1. Walls	noSlip	JohnsonJackParticleSlip SpecularityCoefficient:0.2	Zero gradient	JohnsonJackParticleTheta restitutionCoefficient 0.95 specularity coefficient 0.2
2. Gas inlet	fixedValue Uniform 0.75 m/s or 1 m/s	Fixed value Uniform 0	Zero gradient	Fixed value Uniform 1e-4
3. Gas outlet	pressureInletOutletVelocity	Fixed value Uniform 0	Zero gradient	zero gradient
4. Solid inlet	pressureInletOutletVelocity	Fixed value Uniform 0.315 m/s	Fixed value Uniform 0.63	zero gradient
5. Solid outlet	pressureInletOutletVelocity	fixedValue Uniform 0.04 m/s	Fixed value Uniform 0.63	zero gradient

Among the boundary conditions described in Table 5, the 'pressureInletOutletVelocity' condition is employed on specific boundaries within the computational domain. This condition, widely utilized in OpenFOAM, allows fluid flow to freely enter or exit the computational domain through the respective boundary. It plays a crucial role in capturing the dynamic behavior of the fluid system and its interaction with the surrounding environment.

Additionally, it is worth noting that, for the purpose of validation, we opted to use air instead of methanol vapor in this simulation. This decision allowed us to directly compare our simulation results with experimental data obtained from Particle Image Velocimetry and Digital Image Analysis (PIV-DIA). Such a validation process strengthens the reliability and accuracy of our simulation results and enhances the overall confidence in the numerical model's predictive capabilities.

### Model validation

For model verification, the PIV-DIA technique was used, requiring a transparent Plexiglass setup in two dimensions. The setup consists of a pseudo-2D CFB filled with solid particles of 0.92mm diameter and 1095 kg/m3 density, fluidized with air as shown in Fig. 1. The hydrodynamic features of this setup are consistent with those of the MTO process industrial pilots^4^. Although two-fluid models are still utilized in 2D simulation along with the granular flow, the Gidaspow drag models can provide a good prediction of the solids distribution and velocity field for Goldart B particles used in the experimental test. Therefore, the Gidaspow model was employed to calculate the drag coefficient in the 2D model. The interphase momentum exchange coefficient for the Gidaspow drag model can be calculated using the Eqs. (21–22):

21 $$\beta =\frac{3}{4}\frac{{C}_{D}{\alpha }_{g}{\alpha }_{s}{\rho }_{g}\left|{u}_{g}-{u}_{s}\right|}{{d}_{p}}{{\alpha }_{g}}^{-2.65} ; {\alpha }_{s}<0.2$$

22 $$\beta =150\frac{{\mu }_{s}{{\alpha }_{s}}^{2}}{{{\alpha }_{g}}^{2}{{d}_{p}}^{2}}+1.75\frac{{\rho }_{g}{\alpha }_{s}}{{\alpha }_{g}{d}_{p}}\left|{u}_{g}-{u}_{s}\right|; {\alpha }_{s}>0.2$$

**Figure 5:**
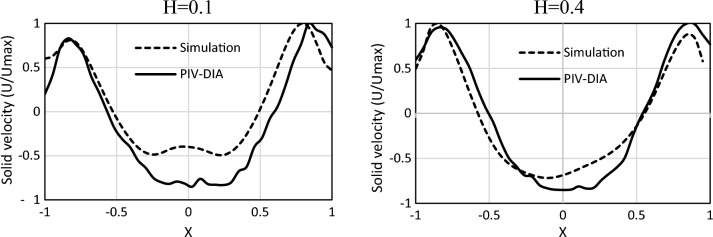
Solid average velocity from simulation and PIV-DIA.

The time-averaged vertical velocity of particle over dimensionless width (X = x/W) at two different heights (H = y/h) of 0.2 and 0.4 is displayed in Fig. 5. The trend of simulated data is similar to that of the experimental results, which demonstrates the potential of the model to be extended to other cases.

## Results and discussion

The investigation of the solids' flow pattern in various geometries was conducted to determine the appropriate gas and solid particle circulation pattern. Subsequently, the results of solid phase volume fraction and granular temperature were analyzed to gain further insights into the mixing characteristics of the circulating fluidized bed used in MTO. The behavior of the bubble phase was also examined by analyzing the diameter of the bubbles.

### Solids flow pattern

The study focused on the velocity field of solid particles in a bubbling fluidized bed. Bubbles introduced in the bed through excess gas are the primary mixing agents. The bed exhibited two symmetrical flow loop regions that play a crucial role in particle mixing, with catalyst particles flowing downwards near the walls and upward motion in the central region. The bottom section showed a continuous discharge of particles toward the standpipe.

**Figure 6:**
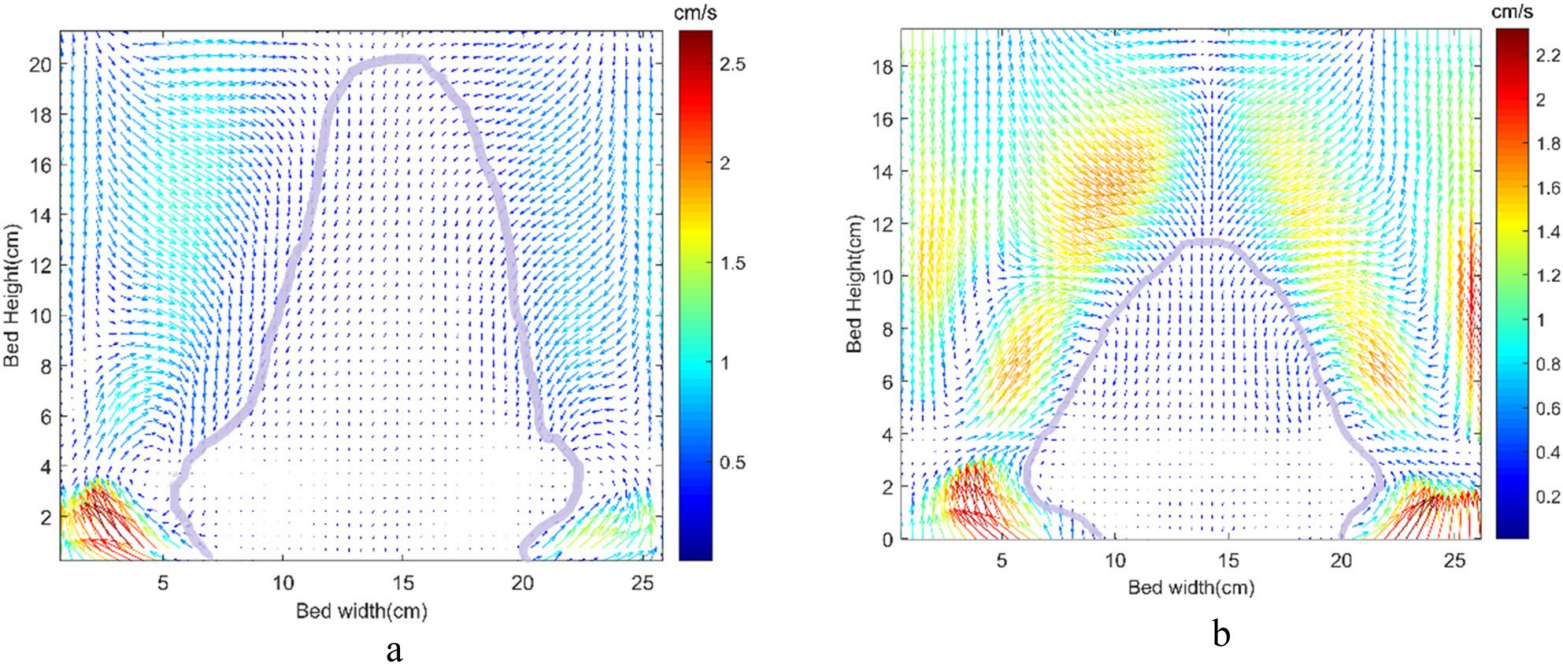
Averaged solid flow pattern from the PIV-DIA method for fluidization velocity in the lower part of the bed at (**a**) 0.75 m/s, and (**b**) 1 m/s (Geom. 1).

Figure 6 illustrates the region with a lower velocity at the bottom of the bed from PIV-DIA data with its highlighted border. As shown, an upward motion of particles can be observed at the left and right sides of the gas distributor, corresponding to the upward gas flow in this section. On the other hand, there is a downward motion of particles at the bed center due to the downward movement of particles toward the standpipe. One of the main observations in our experimental work was the flow of low-velocity particles in the bottom of the bed (See Fig. 6). This plug flow trend of particle movement is not suitable for the mixing of particles. The slow downward movement of particles at the standpipe resists the particle movement in the central region of the bed near the gas distributor, directly affecting the particle mixing inside the bed. This dead region, probably created due to the non-uniform distribution of the gas, is illustrated clearly by the PIV-DIA results. In the context of the methanol-to-olefin (MTO) process, efficient mixing of catalyst particles is crucial for achieving desired conversion and yield. It is important to ensure that the gas reaction uniformly contacts the catalyst particles. In Fig. 6, it can be observed that increasing the gas velocity reduces stagnant regions. However, higher gas velocity also leads to increased gas back-mixing and bubble size, as reported in previous studies^37,38^. This, in turn, results in longer local residence time of gas in the reactor, leading to higher production of undesirable by-products and reduced residence time for gas passing through the bed with larger bubbles. On the other hand, it is important to maintain a gas–solid contact time of around 2 to 3 s in the MTO reaction to avoid the formation of undesired products^2^. The selection of two gas velocities in simulation was done by considering these factors.

To address the issue of low-velocity particles in the bottom of the bed and improve particle mixing, further investigation is needed to optimize the gas distribution and minimize stagnant regions. By implementing strategies that enhance gas–solid contact time without compromising the formation of undesired products, the efficiency of the methanol-to-olefin process can be significantly improved.

To ensure optimal gas velocity within an applicable range for scale-up and industrial applications, we explored potential solutions such as introducing baffles in various configurations. The installation height of the baffles was carefully determined based on a comprehensive analysis of the flow field. Initially, the area with a high particle volume fraction was identified using PIV-DIA (Particle Image Velocimetry—Digital Image Analysis) and subsequently confirmed through simulation. To strategically optimize the effectiveness of the baffles, they were installed at the center of this dense region. This approach aims to disrupt the stagnant flow patterns and enhance the mixing of particles throughout the bed. Further experimentation and analysis will be conducted to assess the impact of these baffles on particle movement and overall reactor performance, with the ultimate goal of achieving improved conversion and yield in the MTO process.

The mixing patterns for different geometries were simulated and visualized in Fig. 8. In Fig. 8a, it can be observed that the presence of a symmetric baffle leads to an appropriate mixing pattern, particularly in the lower part of the bed. By increasing the fluidization velocity, an almost symmetrical pattern is achieved; however, this creates a low-velocity region with inadequate mixing. Comparatively, Fig. 8c demonstrates that a larger asymmetric baffle generates a stronger secondary flow compared to Fig. 8a. At higher fluidization velocities, a slight change in the mixing pattern is observed, as depicted in Fig. 8d. It is crucial for the bed to have a suitable mixing pattern at lower gas velocities to prevent gas back-mixing, as mentioned earlier. However, Fig. 8e shows that a symmetrical pattern creates a low-velocity zone above the baffle, lacking proper mixing. Notably, increasing the fluidization velocity, as illustrated in Fig. 8f., improves the mixing pattern.

**Figure 7:**
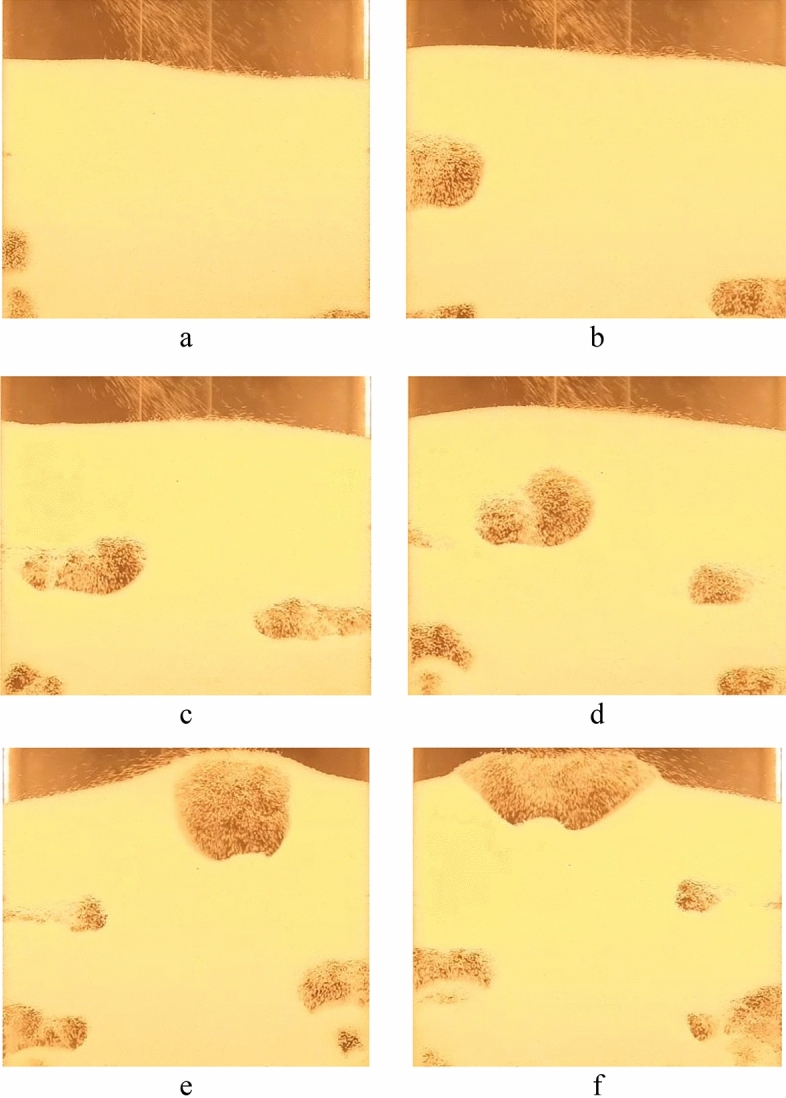
Consequent snapshots from the process of rising bubbles in the bed at a fluidization velocity of 0.75 m/s (Geom. 1).

The upward movement of bubbles in the fluidized bed causes most solid particles to move upwards as well. Eventually, the bubbles merge and disintegrate as they reach the top of the bed, and the particles subsequently move down the walls, as depicted in Fig. 7. This behavior is consistent with the bubbling fluidized bed observed in other references^12,13^.

**Figure 8:**
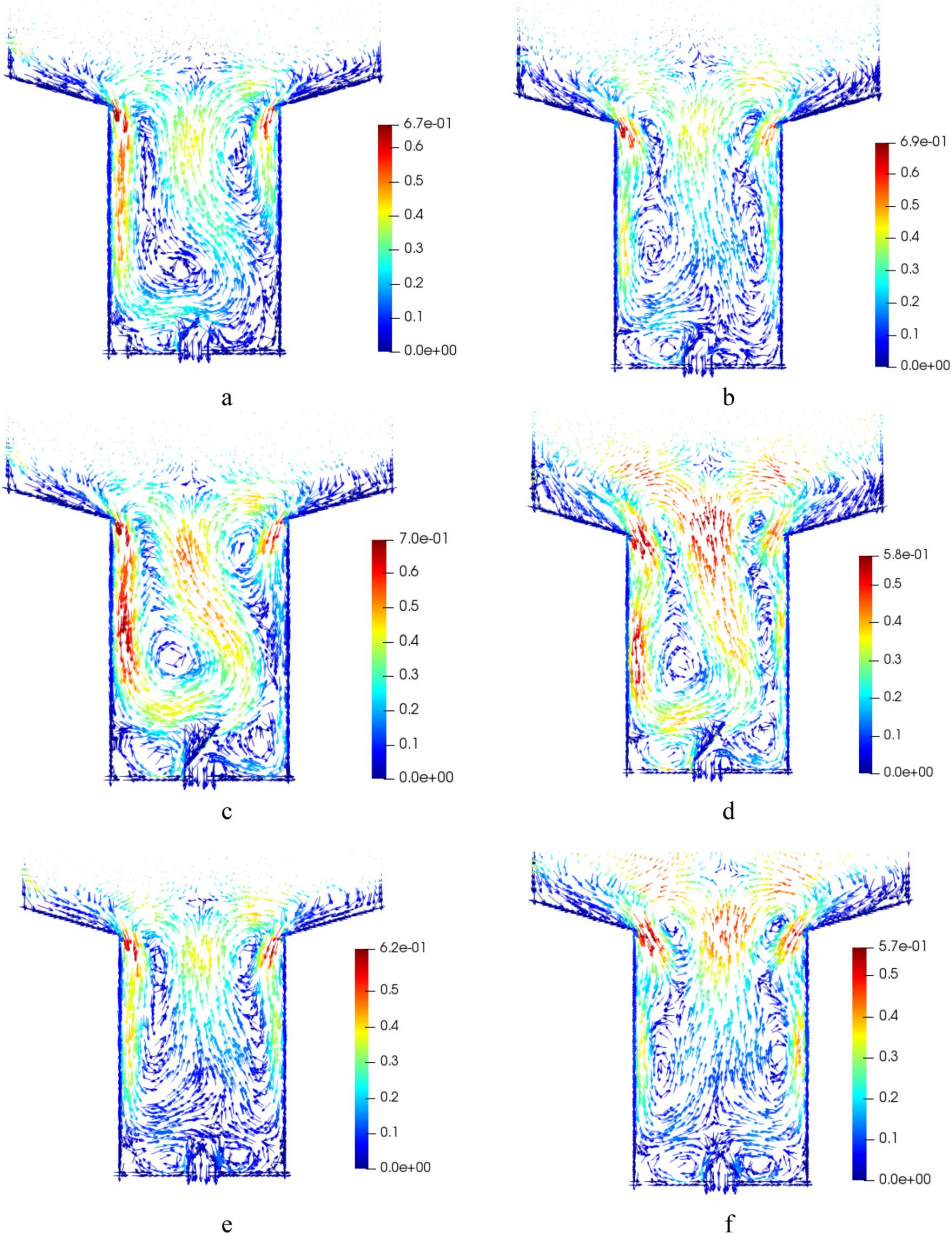
Averaged solid flow pattern from simulation for cases: (**a**) 0.75 m/s, Geom. 2, (**b**) 1 m/s, Geom. 2, (**c**) 0.75 m/s, Geom. 3, (**d**) 1 m/s, Geom. 3, (**e**) 0.75 m/s, Geom. 4, and (**f**) 1 m/s, Geom. 4.

Furthermore, the effect of adding baffles in various configurations was investigated, as shown in Fig. 8. Figure 8a demonstrates that an asymmetric baffle leads to an appropriate mixing pattern, particularly in the lower part of the bed. Increasing the fluidization velocity generates an almost symmetrical pattern, resulting in a low-velocity region with inadequate mixing. Figure 8c reveals that a larger asymmetric baffle induces a stronger secondary flow compared to Fig. 8a. At higher fluidization velocities, the mixing pattern undergoes slight changes, as depicted in Fig. 8d. It is essential to achieve a suitable mixing pattern at lower gas velocities to prevent gas back-mixing, as previously mentioned. However, Fig. 8e shows that an asymmetrical pattern creates a low-velocity zone above the baffle, which lacks proper mixing. Increasing the fluidization velocity improves the mixing pattern, as illustrated in Fig. 8f.

### Volume fraction of solid phase

**Figure 9:**
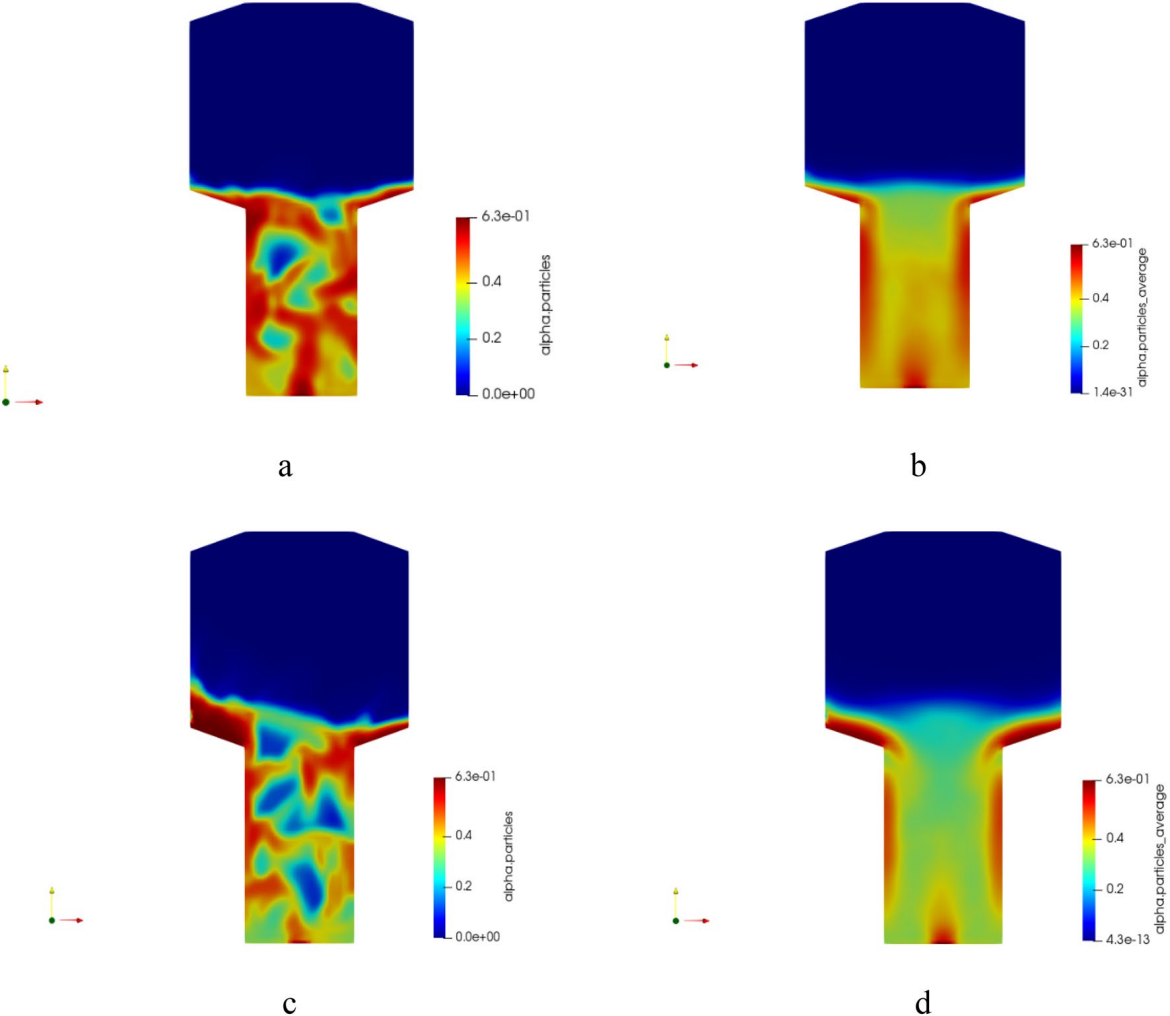
Snapshot and averaged volume fraction of solid phase from simulation for (**a**) Snapshot at t = 150 Sec; u1 = 0.75 m/s, (**b**) Averaged; u2 = 0.75 m/s, (**c**) Snapshot at t = 150 Sec; u2 = 1 m/s, and (**d**) Averaged; u2 = 1 m/s (Geom. 1).

Figure 9 shows the averaged volume fraction of particles in the reaction zone of the MTO reactor, obtained from CFD results for Geom 1. These simulations were conducted employing the two-phase Eulerian–Eulerian model within our framework, enabling us to calculate the solid volume fraction distribution within the reactor geometry, including regions conventionally termed 'bubbles.'

However, it is worth noting that our simulation does not explicitly incorporate parameters pertaining to bubble diameter and coalescence. Instead, our focus remains on understanding the overall behavior of particles within the reactor's reaction zone. The snapshots of the volume fraction plot in Fig. 9 reveal the presence of a bubbling regime within the reaction zone, accompanied by the emergence of a dead zone near the standpipe.

The higher particle volume fraction observed in the middle part of the reactor bottom is due to the standpipe mechanism, which promotes particle migration towards the central region. This localized particle distribution is a characteristic feature of the MTO reactor design and does not indicate any irregularity or malfunction. Rather, it provides insights into the hydrodynamics and particle behavior within the reactor, contributing to a comprehensive understanding of the process.

**Figure 10:**
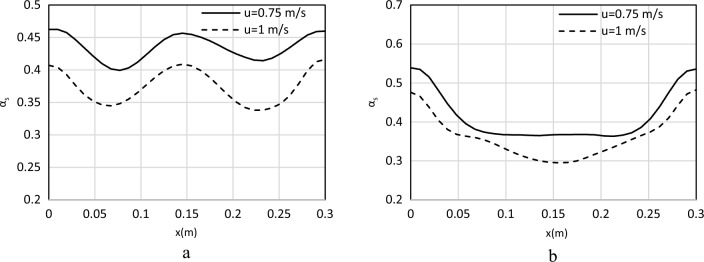
Averaged solid volume fraction profiles for (**a**) h/ht = 0.2, 0.75 m/s, 1 m/s, (**b**) h/ht = 0.8, 0.75 m/s, 1 m/s, (Geom. 1).

The averaged particle volume fraction at the dimensionless heights (h/h_t_) of 0.2 and 0.8 are shown in Fig. 10.

**Figure 11:**
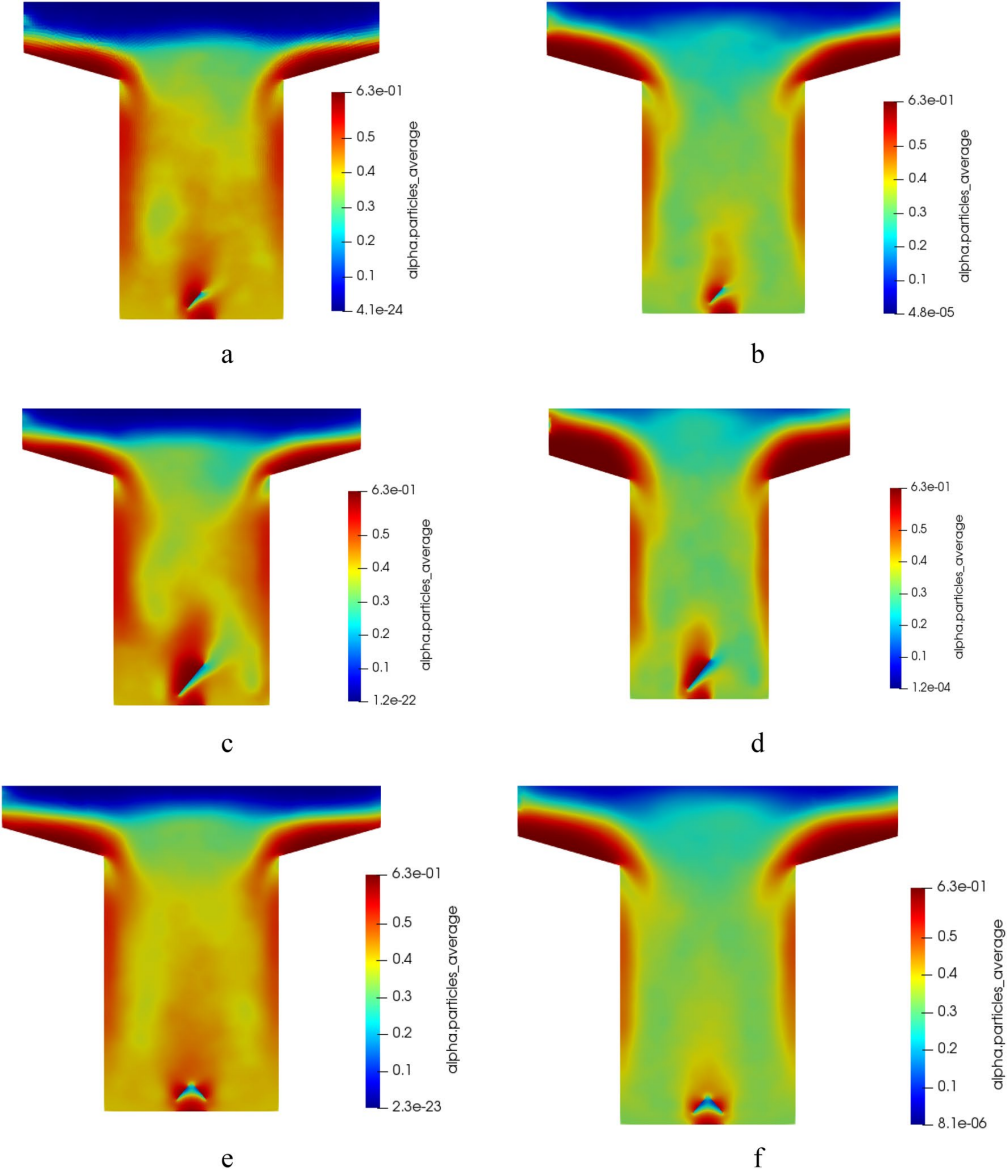
The time-averaged volume fraction of solid-phase from simulation for (**a**) Snapshot at t = 150 Sec; 0.75 m/s, Geom. 2, (**b**) Averaged; 1 m/s, Geom. 2. (**c**) Snapshot at t = 150 Sec; 0.75 m/s, Geom. 3, (**d**) Averaged; 1 m/s, Geom. 3. (**e**) Snapshot at t = 150 Sec; 0.75 m/s, Geom. 4, and (**f**) Averaged; 1 m/s, Geom. 4.

Non-uniform solid flow and improved gas–solid mixing patterns are observed due to the volume fraction changes around the baffles. Figure 11 illustrates the average solid volume fraction of particles in various scenarios. The volume fraction alterations around the baffle are more extensive in Geom. 3 than in other cases.

### Granular temperature

**Figure 12:**
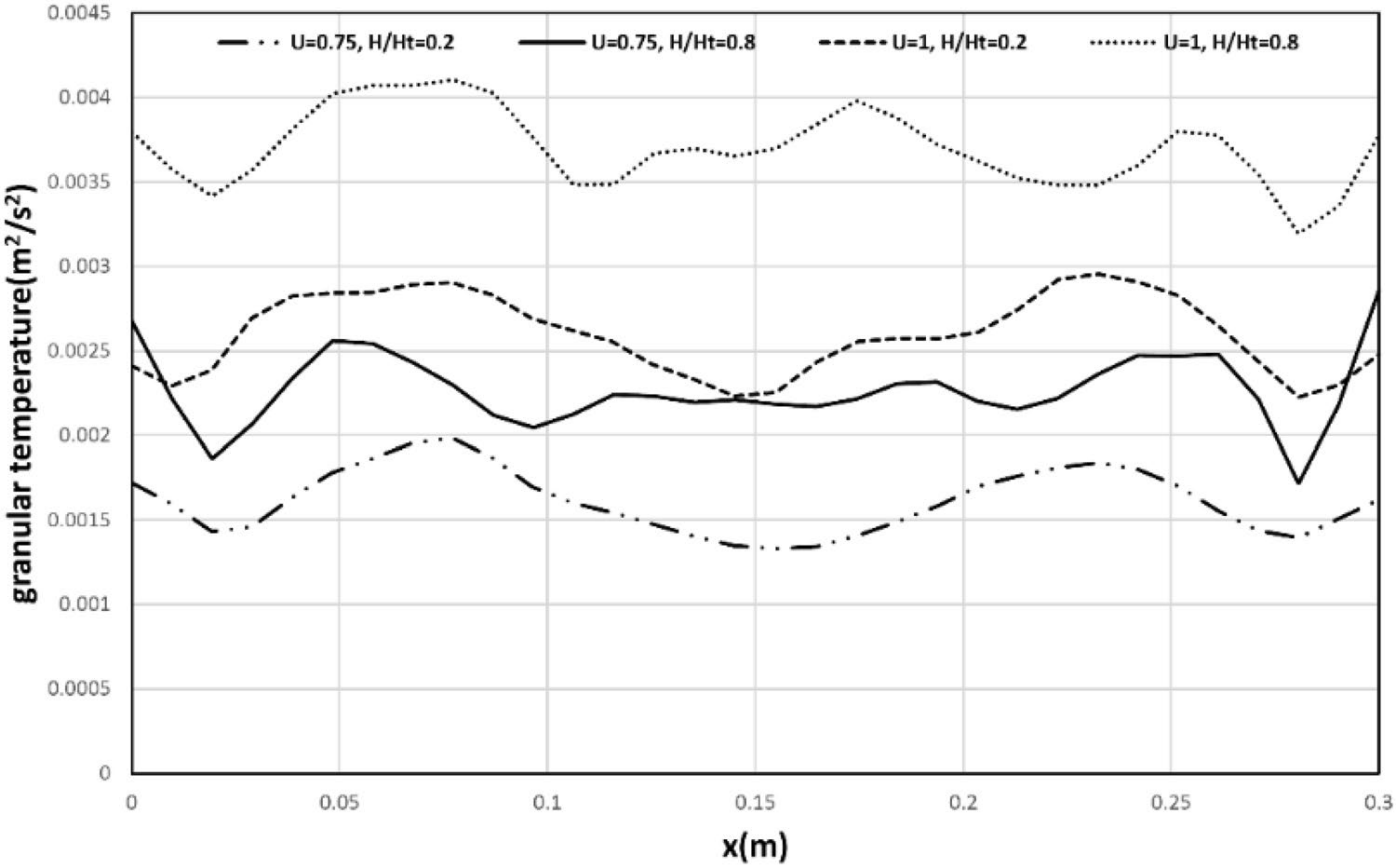
Time-averaged granular temperature from simulation for case of Geom. 1.

The simulation provides an important benefit by allowing for the straightforward calculation of granular temperature, which is difficult to measure experimentally. This temperature reflects the fluctuation of particles in different areas of the bed. Figure 12 illustrates the granular temperature as a function of bed width at various bed heights for Geom. 1. The Fig. 12 shows that the granular temperature has a consistent trend at different bed heights, indicating that the bubble motion is able to generate uniform mixing. Furthermore, an increase in gas velocity results in greater particle oscillation, and consequently, an increase in granular temperature. The granular temperature is observed to increase with an increase in bed height, which can be attributed to the role of bubbles in mixing and particle fluctuation. In the following section, the diameter of bubbles is experimentally determined to provide further insight into their mixing capability. It is worth noting that due to the presence of a dead region at the bottom of the bed, the oscillation of particles at lower heights is less, and thus the granular temperature does not increase significantly.

**Figure 13:**
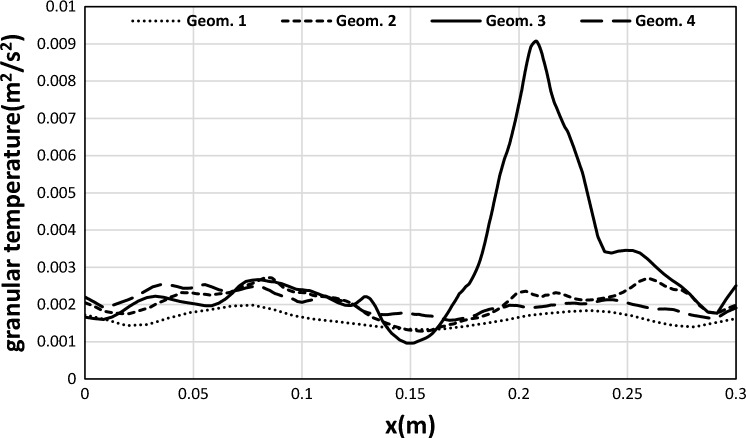
Time-averaged granular temperature from simulation for different cases in h/ht = 0.2 and 0.75 m/s.

Figure 13 shows the effect of the baffle on granular temperature. It can be seen that Geom. 3 has the maximum granular temperature near the upper tip baffle, which can be attributed to the effect of the upper tip baffle in the mixing and fluctuation of solid particles.

### Average bubble diameter

**Figure 14:**
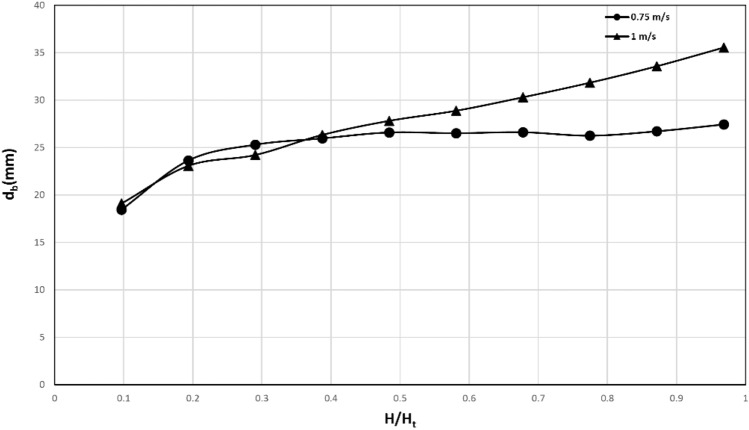
Bubble diameter versus bed height from DIA data (Geom. 1).

Studying the diameter of bubbles is crucial in comprehending their role in the mixing characteristics of the bed. The bubble diameters are calculated from experimental data obtained through the DIA technique. Figure 14 presents the averaged bubble diameter diagram versus the bed height. The bubbles originate from both the left and right sides of the gas distributor. The downward flow of solids to the standpipe, as previously depicted in Fig. 6, creates a pressure drop against the upward movement of bubbles. As a result, the growth of bubbles is hindered as compared to conventional beds. Moreover, the existence of a dead region at the bottom of the bed limits the effect of gas velocity on increasing the bubble diameter. Figure 14 illustrates that the impact of gas velocity on the bubble diameter in the lower part of the bed is minimal. Conversely, larger bubbles are formed as the gas velocity increases from the middle height of the bed. As the bubble diameter expands, the adhesion of particles inside the bubble also increases. However, the height of the bed confines the diameter of the bubbles, causing them to dissociate, and the particles inside the bubbles to return to the bed. This observation aligns with the behavior previously observed in Fig. 7.

## Conclusions

The hydrodynamics of a two-dimensional circulating fluidized bed in the MTO process were thoroughly investigated using a combination of experimental and simulation techniques. This comprehensive study has led to several key findings, significantly contributing to our understanding of the system's behavior.

- The analysis of solid velocity patterns provided valuable insights into the flow behavior within the bed. Specifically, we identified a low-velocity region of particles at the bottom of the bed, indicating poor gas–solid mixing in this area. To validate this observation quantitatively, we conducted an in-depth analysis of particle velocities in the identified region, confirming the presence of inadequate gas–solid interactions. These quantitative insights underscore the significance of proper solid mixing patterns for achieving efficient gas–solid mixing in circulating fluidized beds.
- To further explore the influence of geometrical considerations on mixing performance, we employed simulations with different geometries, including asymmetric and symmetrical baffles near the solid outlet. Through quantitative analyses, such as solid volume fraction and granular temperature assessments, we found that the asymmetric baffle had a more pronounced impact on mixing compared to the symmetrical baffle. This quantitative comparison reveals the importance of selecting appropriate geometrical configurations to optimize the fluidization process.
- Utilizing the DIA method, we quantitatively analyzed the images to evaluate the diameter of bubbles in the bed. Our quantitative assessment identified the presence of a low mixing region, which restricts the bubble diameter. This observation provides a clearer understanding of the impact of hydrodynamics on bubble formation and size, offering insights into potential improvements in bubble behavior within the bed.
- To ensure the accuracy and validity of our simulations, we conducted a comprehensive comparison of predictions from different geometries and flow conditions. we rigorously compared key parameters, including particle velocities, gas–solid mixing efficiencies, and pressure drop variations. This comparison allowed us to gain deeper insights into the performance of each scenario, guiding us towards valuable conclusions regarding the bed's hydrodynamics.

In conclusion, this study significantly enhances our knowledge of circulating fluidized beds in the MTO process. The incorporation of data and rigorous analysis strengthens the conclusions drawn, providing robust evidence to support our findings. The insights gained from this research have the potential to optimize the design and operation of circulating fluidized beds in various industrial applications.

## Data Availability

Data are available with the permission of [Salman Movahedirad]. The data that support the findings of this study are available from the corresponding author, upon reasonable request.

## List of symbols

${C}_{D}$: Drag coefficient (dimensionless)
$${d}_{p}$$: Particles diameter (m)
${e}_{s}$: Restitution coefficient (dimensionless)
${f}_{s}$: Granular conductivity (Kg/m s)
$GSV$: Gray-scale value of a pixel (dimensionless)
$g$: The subindex for the gas phase
$\mathrm{g}$: Acceleration of gravity (m/s^2^);
${g}_{0}$: Radial distribution (dimensionless)
I: Identity matrix (dimensionless)
${J}_{slip}$: Granular temperature production due to fluid turbulence (Kg/m s^3^)
${J}_{vis}$: Dissipation of granular energy due to viscous damping (Kg/m s^3^)
$$\mathrm{p}$$: Pressure (Pa)
${p}_{s}$: Solids pressure (Pa)
${Re}_{p}$: Particle Reynolds number (dimensionless)
$s$: The subindex for the solid phase
$$u$$: Phase velocity (m/s)

## Greek symbols

$\alpha$: Phase volume fraction (dimensionless)
${\left({\alpha }_{s}\right)}_{ref}$: The volume fraction of the solid phase in reference mode (dimensionless)
$\beta$: Momentum transfer coefficient^’^ drag (Kg/m^3^ s)
${\gamma }_{s}$: Granular temperature dissipation due to inelastic collisions (Kg/m s^3^)
$$\theta $$: Granular temperature (m^2^ /s^2^)
$\lambda$: Bulk viscosity (Pa s)
$\mu$: Dynamic viscosity (Pa s)
$\rho$: Phase density (Kg/ m^3^)
$\tau$: Shear stress tensor (N/m^2^)
